# An Investigation Into the Hepatoprotective Properties of Berberis asiatica, Withania somnifera, and Their Combination in Streptozotocin-Nicotinamide-Induced Type II Diabetes Mellitus in Wistar Rats

**DOI:** 10.7759/cureus.78690

**Published:** 2025-02-07

**Authors:** Devkumar D Tiwari, Vandana M Thorat, Prathamesh V Pakale

**Affiliations:** 1 Department of Pharmacology, Krishna Institute of Medical Sciences, Krishna Vishwa Vidyapeeth (Deemed to be University), Karad, IND; 2 Department of Pharmacology and Therapeutics, Krishna Institute of Medical Sciences, Krishna Vishwa Vidyapeeth (Deemed to be University), Karad, IND

**Keywords:** experimental animal model, hepatoprotective, histopathology examination, lft, type 2 diabetes mellitus (t2dm)

## Abstract

Background: The rising prevalence of type II diabetes mellitus (T2DM) has increased the risk of hepatic dysfunction, necessitating effective therapeutic interventions. Natural products, particularly *Berberis asiatica* (BA) and *Withania somnifera* (WS), are known for their hepatoprotective properties.

Objectives: This study investigates the hepatoprotective effects of BA, WS, and their polyherbal combination in streptozotocin-nicotinamide-induced T2DM in Wistar rats.

Methods: Seventy-eight adult albino Wistar rats were divided into 13 groups, including normal control, disease control, and treatment groups, which received BA, WS, and a polyherbal combination (PHC) at varying doses (250, 500, and 1,000 mg/kg). Metformin and glimepiride served as standard treatments. Liver function was assessed through serum glutamic pyruvic transaminase (SGPT) and bilirubin levels, and histopathological analysis was performed.

Results: Treatment with BA, WS, and PHC significantly reduced SGPT and bilirubin levels compared to the disease control (p < 0.001). The PHC demonstrated superior efficacy, with higher doses (1,000 mg/kg) exhibiting results comparable to standard drugs. Histopathological analysis revealed reduced hepatocellular damage in treated groups, indicating preserved hepatic architecture.

Conclusion: BA and WS, individually and in combination, significantly attenuated liver dysfunction in diabetic rats. The synergistic effect of PHC presents a promising natural therapeutic approach for managing diabetes-induced liver damage.

## Introduction

The global prevalence of type II diabetes mellitus (T2DM) has reached alarming levels, increasing from 537 million adults in 2021 to 643 million in 2030 and 783 million in 2045. This poses significant challenges to public health systems worldwide [1]. Characterized by insulin resistance and progressive pancreatic β-cell dysfunction, T2DM is associated with a host of complications, including hepatic dysfunction and nephropathy [2]. Diabetes-induced liver damage, often termed diabetic hepatopathy, is marked by elevated hepatic enzymes and altered metabolic pathways, making the liver a critical organ in the pathology of diabetes [3]. Similarly, the role of oxidative stress and inflammation in diabetic nephropathy has garnered significant attention, emphasizing the need for therapeutic interventions targeting multiple pathways [4].

The liver’s central role in glucose metabolism and detoxification makes it particularly susceptible to damage in T2DM. Chronic hyperglycemia leads to nonalcoholic fatty liver disease, inflammation, and fibrosis, which are exacerbated by oxidative stress and dyslipidemia [5]. The pathophysiology of diabetic hepatopathy involves mitochondrial dysfunction, lipid peroxidation, and the overproduction of reactive oxygen species [6]. Elevated serum glutamic pyruvic transaminase (SGPT) and bilirubin levels often signify liver damage in diabetic models, highlighting the need for hepatoprotective agents [7].

Exploring plant-derived compounds as hepatoprotective agents has gained momentum due to their multitargeted mechanisms, minimal side effects, and affordability. Among these, *Berberis asiatica* (BA) and *Withania somnifera* (WS) have shown remarkable pharmacological properties. BA, a member of the Berberidaceae family, contains bioactive compounds such as berberine and alkaloids known for their anti-inflammatory and antioxidant activities. WS, commonly known as ashwagandha, is a renowned adaptogen with bioactive withanolides that enhance antioxidant defenses and modulate stress responses [8].

The primary aim of this study was to evaluate the hepatoprotective effects of BA and WS, both individually and in combination, in mitigating diabetes-induced liver dysfunction. Future research will focus on conducting clinical trials in human subjects to validate these hepatoprotective effects and establish optimal dosing regimens. Additionally, we aim to explore the molecular mechanisms underlying the synergistic interactions between BA and WS, providing deeper insights into their therapeutic potential. Investigating their role in other metabolic disorders linked to oxidative stress and inflammation, such as diabetic nephropathy and cardiovascular diseases, will further extend the scope of this research in integrative medicine.

## Materials and methods

Experimental animals

The animals used for this study were albino Wistar adult rats of either sex (male/female) weighing 150-250 g and 8-12 weeks old. Rats were procured from the Central Animal House of Krishna Institute of Medical Sciences, Krishna Vishwa Vidyapeeth (KVV), Satara, India.

Facilities and equipment

Animals were kept in a standardized air-conditioned laboratory, maintaining 12 hours of natural dark-light cycles at the atmospheric temperature of 27°C-37°C. They had free access to standard diet and water as desired until the end of the study. Commercial pellet diet contained 55% carbohydrates, 24% protein, 10% moisture, 5% fat, 4% fiber, 0.6% calcium, 0.3% phosphorous, and 9% ash w/w from VRK solutions, Sangali, India. About three to four rats were housed in cages made up of polyacrylic material (40 × 25 ×15).

Ethical aspects and compliance

Ethical approval was taken from the Institutional Ethics Committee (IEC) of KVV (IEC approval no. 385/2020-2021), and animal ethical approval was taken from the Institutional Animal Ethics Committee of KVV, reg. no. 255/PO/REBi/S/2000/CPCSEA (IAEC approval no. IAEC/KIMS/2021/16).

All research was carried out in accordance with the guidelines of the Committee for Control and Supervision of Experiments on Animals at the Central Animal House, KVV, Karad.

Chemical and drugs

All the chemicals, drugs, and reagents utilized in the research were of analytical quality. The polyherbal combination (PHC) of the BA and WS in the ratio of 1:1 was used for this study.

Drugs Used for Induction of T2DM

Streptozotocin (STZ) and nicotinamide (NIC) in pure powdered form were purchased from Sisco Research Laboratories Pvt. Ltd., Mumbai, India.

Test Drugs

The standardized dried ethanolic root extracts of BA and WS in pure powdered form were purchased from Natucare India Pvt. Ltd., Thane, India, and Bhagwati Herbal and Healthcare Pvt. Ltd., Vapi, India.

Standard Drugs

Metformin (MET) and glimepiride (GLI) in pure powdered form were purchased from Smruti Organics Limited, Solapur, India.

Preparation of reagents

STZ

Sixty-five milligrams per kilogram body weight of STZ was prepared by dissolving it in a freshly prepared cold citrate buffer 0.1 M with a pH of 4.5.

NIC

One hundred ten milligrams per kilogram body weight of NIC was freshly prepared by dissolving in 0.9% physiological saline.

Citrate Buffer (0.1 M)

The citrate buffer was prepared using citric acid (10.5 g) and sodium citrate (14.7 g). These reagents were weighed and mixed in 500 mL of water [9]. The final volume was made up to 1 L with distilled water (d/w). The pH was adjusted by hydrochloric acid or sodium hydroxide to 4.5.

Experimental groups and procedure

Seventy-eight adult albino Wistar rats were randomly divided into the following 13 experimental groups, with six rats in each group, and maintained for five weeks.

Group 1: Normal Control

For 28 days, rats were administered d/w orally at a dose of 10mL/kg using a feeding cannula.

Group 2: Disease Control

Rats were injected with STZ-NIC intraperitoneally (i.p.) to induce diabetes at zero weeks and administered d/w at a dose of 10 mL/kg orally using a feeding cannula for 28 days.

Group 3: BA 250

Rats were injected with STZ-NIC i.p. to induce diabetes at zero weeks and received dried ethanolic root extract of BA dissolved in d/w at a dose of 250 mg/kg orally for 28 days.

Group 4: BA 500

Rats were injected with STZ-NIC i.p. to induce diabetes at zero weeks and received dried ethanolic root extract of BA dissolved in d/w at a dose of 500 mg/kg orally for 28 days.

Group 5: BA 1000

Rats were injected with STZ-NIC i.p. to induce diabetes at zero weeks and received dried ethanolic root extract of BA dissolved in d/w at a dose of 1,000 mg/kg orally for 28 days.

Group 6: WS 250

Rats were injected with STZ-NIC i.p. to induce diabetes at zero weeks and received dried ethanolic root extract of WS dissolved in d/w at a dose of 250 mg/kg orally for 28 days.

Group 7: WS 500

Rats were injected with STZ-NIC i.p. to induce diabetes at zero weeks and received dried ethanolic root extract of WS dissolved in d/w at a dose of 500 mg/kg orally for 28 days.

Group 8: WS 1000

Rats were injected with STZ-NIC i.p. to induce diabetes at zero weeks and received dried ethanolic root extract of WS dissolved in d/w at a dose of 1,000 mg/kg orally for 28 days.

Group 9: PHC 250

Rats were injected with STZ-NIC i.p. to induce diabetes at zero weeks and received dried ethanolic root extract of BA+WS dissolved in d/w at a dose of 250 mg/kg orally for 28 days.

Group 10: PHC 500

Rats were injected with STZ-NIC i.p. to induce diabetes at zero weeks and received dried ethanolic root extract of BA+WS dissolved in d/w at a dose of 500 mg/kg orally for 28 days.

Group 11: PHC 1000

Rats were injected with STZ-NIC i.p. to induce diabetes at zero weeks and received dried ethanolic root extract of BA+WS dissolved in d/w at a dose of 1,000 mg/kg orally for 28 days.

Group 12: MET

Rats were injected with STZ-NIC i.p. to induce diabetes at zero weeks and received MET dissolve in d/w at a dose of 250 mg/kg orally for 28 days [8].

Group 13: GLI

Rats were injected with STZ-NIC i.p. to induce diabetes at zero weeks and received MET dissolve in d/w at a dose of 10 mg/kg orally for 28 days [8].

The experimental model of DM

STZ-NIC Diabetic Rat Model

Rakieten et al. reported the diabetogenic activity of the compound STZ, which is specifically cytotoxic to the pancreatic beta cells [10,11]. Masiello et al. crafted a very useful STZ-NIC type 2 diabetes model that demonstrated the (STZ-NIC) based on NIC's potential to provide partial protection against STZ's β-cytotoxic effects [12].

Treatment protocol

The treatment protocol has been presented in Table 1.

**Table 1:** Treatment protocol NC: normal control; DC: diabetic control; BA: *B. asiatica*; WS: *W. somnifera*; PHC: polyherbal combination; MET: metformin; GLI: glimepiride

Group no.	Group name	Extract/drugs	Dose and route (orally), mg/kg
1	NC	Distilled water	10 mL/kg
2	DC	Distilled water	10 mL/kg
3	BA 250	Dried ethanolic root extract of BA	250
4	BA 500	Dried ethanolic root extract of BA	500
5	BA 1000	Dried ethanolic root extract of BA	1,000
6	WS 250	Dried ethanolic root extract of WS	250
7	WS 500	Dried ethanolic root extract of WS	500
8	WS 1000	Dried ethanolic root extract of WS	1,000
9	PHC 250	Dried ethanolic root extract of BA + WS	125 + 125
10	PHC 500	Dried ethanolic root extract of BA + WS	250 + 250
11	PHC 1000	Dried ethanolic root extract of BA + WS	500 + 500
12	MET	Metformin (standard)	250
13	GLI	Glimepiride (standard)	10

Evaluation parameters

Biochemical Parameters

Blood samples from rats of all experimental groups were collected using the heart puncture method at the end of the study when they were sacrificed under light anesthesia. The serum was analyzed using spectrophotometric enzymatic assays. SGPT levels indicated hepatocellular injury, while bilirubin levels reflected bile metabolism.

Histopathological Examination

At the end of the experiment, the rats were sacrificed. The liver and kidney were immediately fixed in a 10% buffered neutral formalin solution. The tissues were carefully embedded in molten paraffin with the help of metallic blocks, covered with flexible plastic molds, and kept under freezing plates to allow the paraffin to solidify. Cross sections (5 mm thick) of the fixed tissues were cut. These sections were stained with hematoxylin and eosin and visualized under a microscope to study the microscopic architecture of the tissues. The investigators performing the histological evaluation will be blind to biochemical results and to treatment allocation.

Statistical analysis

All the results were represented as mean ± standard deviation. Statistical analysis was performed using one-way analysis of variance (ANOVA) followed by a Tukey-Kramer multiple comparison test for post hoc analysis to find the difference between the means. A p value of less than 0.05 was considered statistically significant. P represents the probability factor. All the statistical methods were carried out using the Statistical Package for the Social Sciences software version 29.0.0 (IBM Corp., Armonk, NY).

## Results

Ordinary ANOVA followed by the Tukey-Kramer multiple comparison test (post hoc) revealed statistically significant differences in both the liver function SGPT and bilirubin in all the study groups: normal control, NC, DC, BA 250, BA 500, BA 1000, MET, and GLI (p < 0.0001) (see Table 2).

**Table 2:** Effect of BA on liver functions in rats All the values are expressed in mean ± SD. p < 0.05 is considered significant ^a^DC differs significantly from BA 250, BA 500, BA 1000, MET, and GLI groups ^b^MET differs significantly from DC, BA 250, BA 500, BA 1000, and GLI groups ^c^GLI differs significantly from DC, BA 250, BA 500, BA 1000, and MET groups p < 0.0001 represents statistical significance SGPT: serum glutamic pyruvic transaminase; NC: normal control; DC: diabetic control; BA: *B. asiatica*; MET: metformin; GLI: glimepiride; SD: standard deviation; F: f statistic

Groups	NC	DC	BA 250	BA 500	BA 1000	MET	GLI	F	p
Test									
SGPT (U/L)	55.45 ± 0.68^a^	81.23 ± 0.9^bc^	76.83 ± 1.36^abc^	74.42 ± 1.0^abc^	71.2 ± 0.65^abc^	62.75 ± 0.39^a^	62.35 ± 0.35^a^	730.64	<0.0001
Bilirubin (mg/dL)	0.47 ± 0.05^a^	0.94 ± 0.05^bc^	0.66 ± 0.03^abc^	0.65 ± 0.01^abc^	0.61 ± 0.01^abc^	0.51 ± 0.01^a^	0.51 ± 0.01^a^	180.49	<0.0001

**Figure 1:**
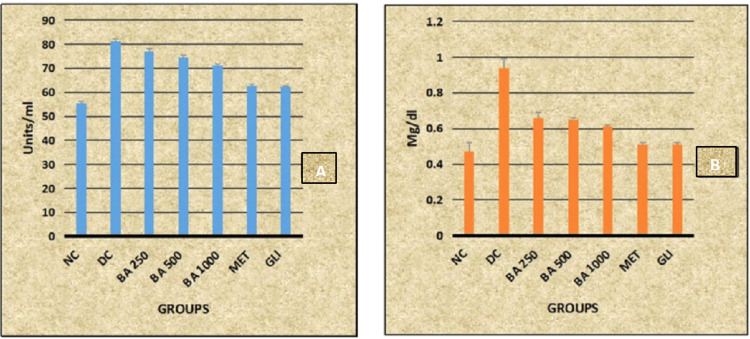
Graphical representation of effect of BA on (A) SGPT and (B) bilirubin level in rats SGPT: serum glutamic pyruvic transaminase; NC: normal control; DC: diabetic control; BA: *B. asiatica*; MET: metformin; GLI: glimepiride

Post hoc analysis revealed statistically significant decreased levels of SGPT and bilirubin in all the study groups (NC, BA 250, BA 500, BA 1000, MET, and GLI) when compared with the DC group (p < 0.001). Whereas statistically significant decreased levels of SGPT and bilirubin are observed in BA 250, BA 500, and BA 1000 groups when compared with MET and GLI groups (p < 0.001) (Table 2, Figure 1).

Histopathological examination of the effect of BA on liver tissue in control groups

NC Liver

**Figure 2:**
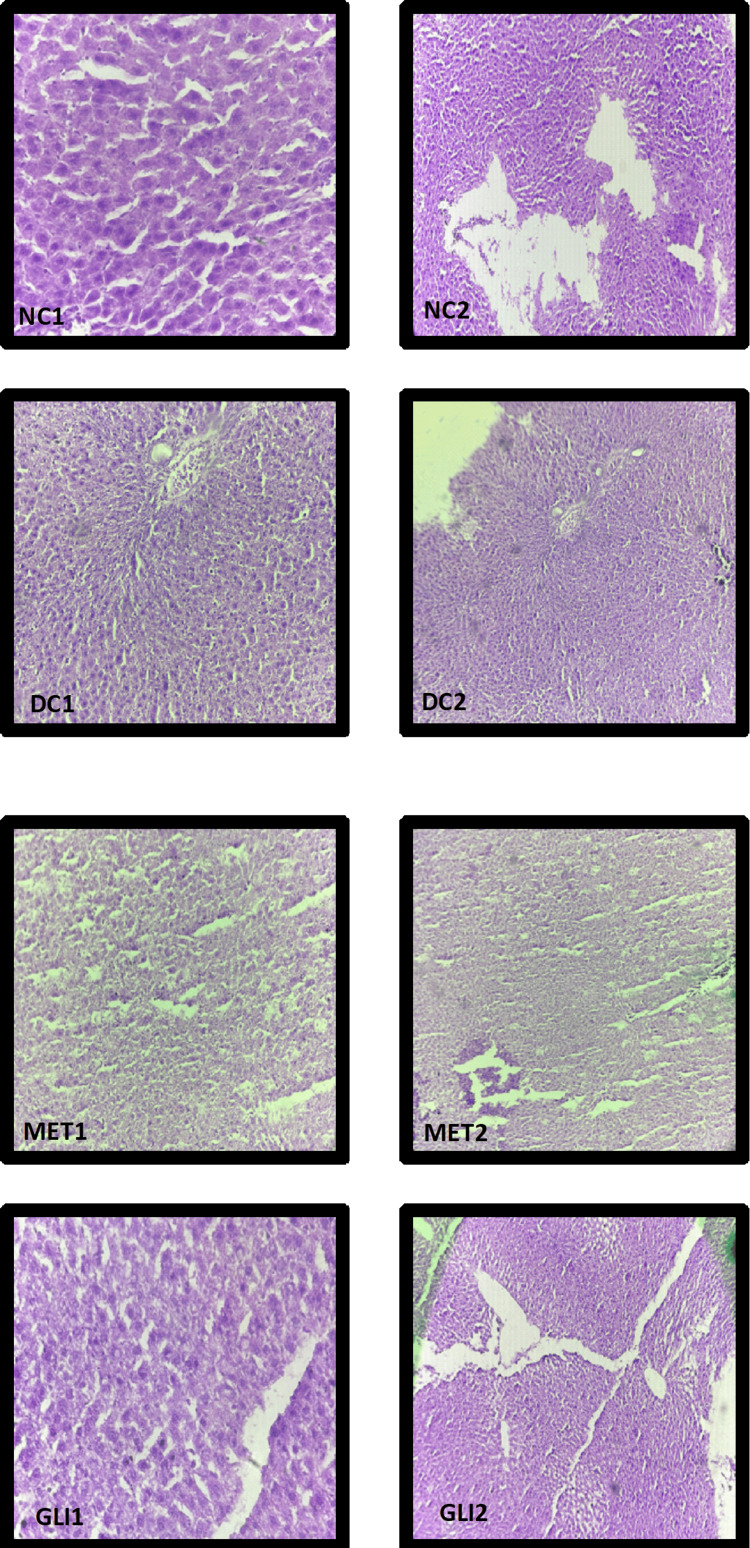
Histopathological examination of effect of BA on liver tissue in control groups BA: *B. asiatica*; NC: normal control; DC: diabetic control; MET: metformin; GLI: glimepiride 1: magnification of 40×; 2: magnification of 10×

Histopathological analysis of the liver cells in the NC group revealed intact hepatic architecture. Central and peripheral veins maintained structural integrity, and hepatocytes showed consistent morphology without signs of congestion, inflammation, or degeneration (Figure 2).​​​​​

DC Liver

Histopathological analysis of the liver cells in the diabetic control (DC) group revealed marked pathological changes, characterized by severe vascular congestion in the central vein and extensive ballooning degeneration of hepatocytes, suggesting significant hepatic damage and cellular distress (Figure 2).

MET Liver

Histopathological analysis of the liver cells in the standard met group revealed mild vascular congestion, reflecting preservation of liver integrity similar to the NC group (Figure 2).

GLI Liver

Histopathological analysis of the liver cells in the standard GLI group revealed normal hepatic architecture, reflecting preservation of liver integrity similar to the NC group (Figure 2).

Histopathological examination of the effect of BA in different doses on liver tissue

BA 250 Liver

**Figure 3:**
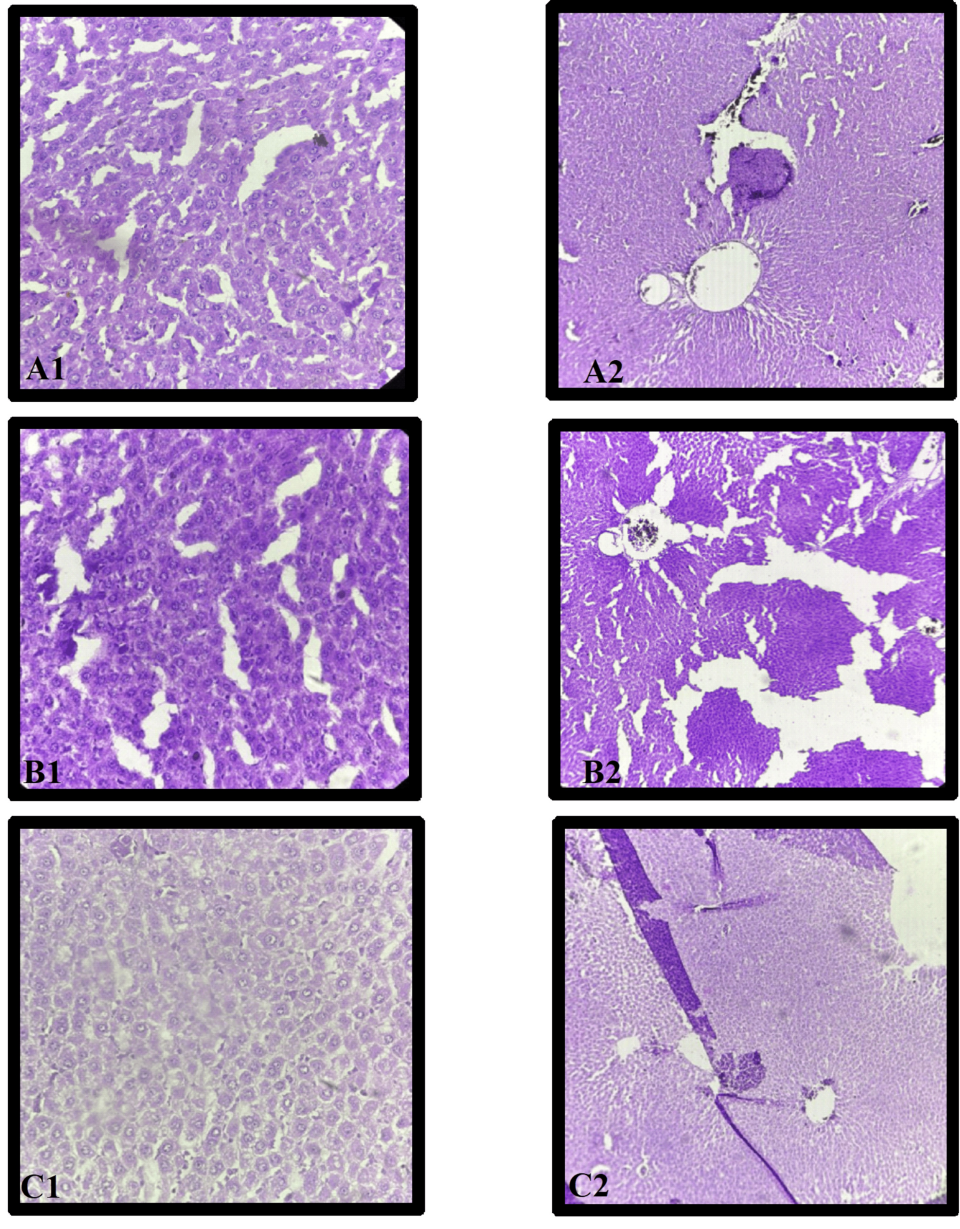
Histopathological examination of effect of BA in different doses on liver tissue BA: *B. asiatica*; A: BA 250; B: BA 500; C: BA 1000 1: magnification of 40×; 2: magnification of 10×

Histopathological analysis of the liver cells in the BA 250 group revealed mild congestion of blood vessels within the central vein, as observed in the photomicrograph under 10× (A2) and 40× (A1) magnifications (Figure 3).

BA 500 Liver

Histopathological analysis of the liver cells in the BA 500 group revealed mild congestion of blood vessels within the central vein, as observed in the photomicrograph under magnification of 10× (B2) and magnification of 40× (B1) (Figure 3).

BA 1000 Liver

Histopathological analysis of the liver cells in the BA 1000 group revealed the normal architecture of the central vein, peripheral vein, and hepatocytes, comparable to that of the NC group, as observed in the photomicrograph under both 10× (C2) and 40× (C1) magnifications (Figure 3).

Ordinary ANOVA followed by the Tukey-Kramer multiple comparison test (post hoc) revealed statistically significant differences in both the liver function SGPT and bilirubin in all the study groups: NC, DC, BA 250, BA 500, BA 1000, MET, and GLI (p < 0.001) (Table 3).

**Table 3:** Effect of WS on liver functions in rats All the values are expressed in mean ± SD. p < 0.05 is considered significant ^a^DC differs significantly from WS 250, WS 500, WS 1000, MET, and GLI groups ^b^MET differs significantly from DC, WS 250, WS 500, WS 1000, and GLI groups ^c^GLI differs significantly from DC, WS 250, WS 500, WS 1000, and MET groups p < 0.0001 represents statistical significance SGPT: serum glutamic pyruvate transaminase; NC: normal control; DC: diabetic control; WS: *W. somnifera*; MET: metformin; GLI: glimepiride; SD: standard deviation; F: f statistic

Group	NC	DC	WS 250	WS 500	WS 1000	MET	GLI	F	p
Test									
SGPT (U/L)	55.45 ± 0.68	81.23 ± 0.9^bc^	75.08 ± 3.00^abc^	73.18 ± 0.84^abc^	70.07 ± 0.58^abc^	62.75 ± 0.39^a^	62.35 ± 0.35^a^	281.88	<0.0001
Bilirubin (mg/dL)	0.47 ± 0.05	0.94 ± 0.05^bc^	0.67 ± 0.02^abc^	0.65 ± 0.01^abc^	0.61 ± 0.01^abc^	0.51 ± 0.01^a^	0.51 ± 0.01^a^	203	<0.0001

**Figure 4:**
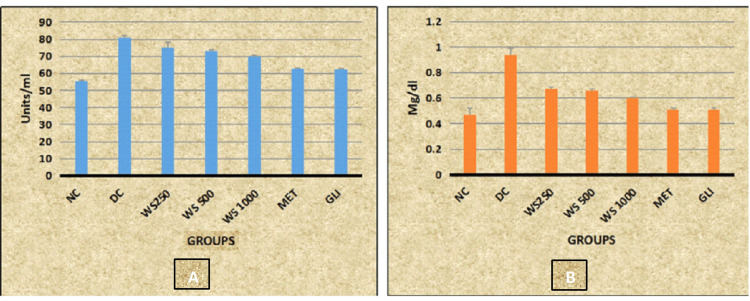
Graphical representation of effect of WS on (A) SGPT and (B) bilirubin level in rats SGPT: serum glutamic pyruvic transaminase; NC: normal control; DC: diabetic control; WS: *W. somnifera*; MET: metformin; GLI: glimepiride

Post hoc analysis revealed statistically significant decreased levels of SGPT and bilirubin in all the study groups (NC, WS 250, WS 500, WS 1000, MET, and GLI) when compared with the DC group (p < 0.001). Whereas statistically significant decreased levels of SGPT and bilirubin are observed in WS 250, WS 500, and WS 1000 groups when compared with MET and GLI groups (p < 0.001) (Table 3, Figure 4).

Histopathological examination of the effect of WS 250 in different doses on liver tissue

WS 250 Liver

**Figure 5:**
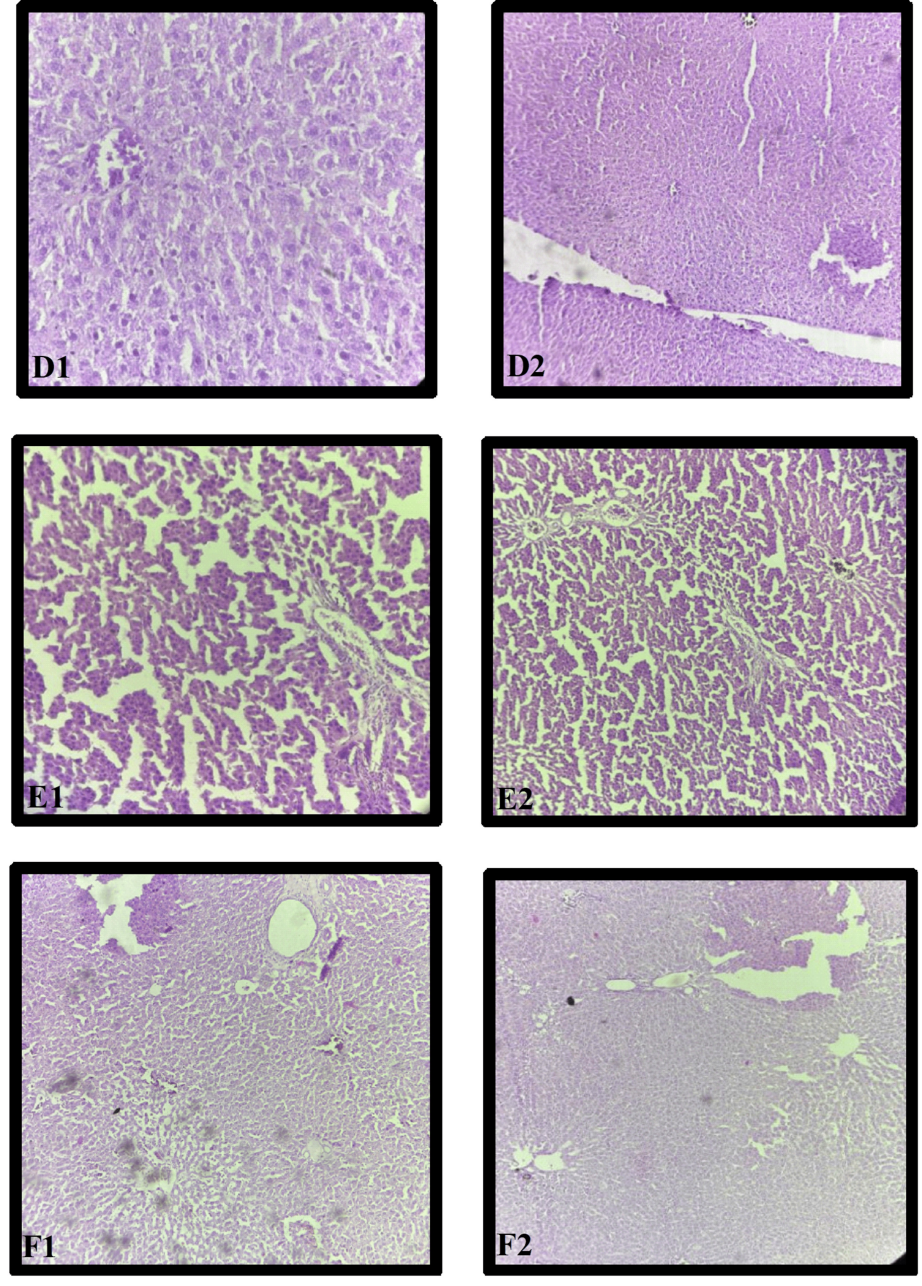
Histopathological examination of effect of WS in different doses on liver tissue D: WS 250; E: WS 500; F: WS 1000; WS: *W. somnifera* 1: magnification of 40×; 2: magnification of 10×

Histopathological analysis of liver cells in the WS 250 group revealed mild congestion of blood vessels within the central vein and focal ballooning degeneration of the cell, as observed in the photomicrograph under both 10× (D2) and 40× (D1) magnifications (Figure 5).

WS 500 Liver

Histopathological analysis of liver cells in the WS 250 group revealed mild congestion of blood vessels within the central vein and focal ballooning degeneration of the cells, as observed in the photomicrograph under both 10× (E2) and 40× (E1) magnifications (Figure 5).

WS 1000 Liver

Histopathological analysis of the liver cells in the WS 1000 group revealed the normal architecture of the central vein, peripheral vein, and hepatocytes, comparable to that of the NC group, as observed in the photomicrograph under both 10× (F2) and 40× (F1) magnifications (Figure 5).

Ordinary ANOVA followed by the Tukey-Kramer multiple comparison test (post hoc) revealed statistically significant differences in both the liver function SGPT and bilirubin in all the study groups (NC, DC, PHC 250, PHC 500, PHC 1000, MET, and GLI) (p < 0.001) (Table 4).

**Table 4:** Effect of PHC on liver functions in rats All the values are expressed as mean ± SD. p < 0.05 is considered significant ^a^DC differs significantly from PHC 250, PHC 500, PHC 1000, MET, and GLI groups ^b^MET differs significantly from DC, PHC 250, PHC 500, PHC 1000, and GLI groups ^c^GLI differs significantly from DC, PHC 250, PHC 500, PHC 1000, and MET groups p < 0.0001 represents statistical significance SGPT: serum glutamic pyruvate transaminase; NC: normal control; DC: diabetic control; PHC: polyherbal combination; MET: metformin; GLI: glimepiride; SD: standard deviation; F: f statistic

Group	NC	DC	PHC 250	PHC 500	PHC 1000	MET	GLI	F	p
Test									
SGPT (U/L)	55.45 ± 0.68	81.23 ± 0.94^bc^	70.93 ± 0.51^abc^	65.63 ± 0.67^abc^	59.65 ± 3.18^abc^	62.75 ± 0.39^a^	62.35 ± 0.35^a^	241.12	<0.0001
Bilirubin (mg/dL)	0.47 ± 0.05	0.94 ± 0.05^bc^	0.64 ± 0.01^abc^	0.59 ± 0.03^abc^	0.50 ± 0.02^a^	0.51 ± 0.01^a^	0.51 ± 0.01^a^	189.62	<0.0001

**Figure 6:**
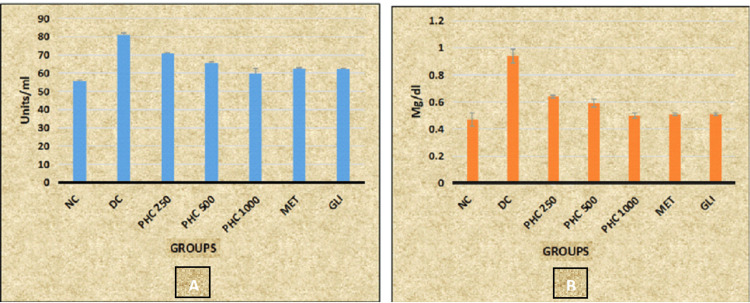
Graphical representation of the effect of PHC on (A) SGPT and (B) bilirubin level in rats SGPT: serum glutamic pyruvic transaminase; NC: normal control; DC: diabetic control; PHC: polyherbal combination; MET: metformin; GLI: glimepiride

Post hoc analysis revealed statistically significant decreased levels of SGPT and bilirubin in all the study groups (NC, PHC 250, PHC 500, PHC 1000, MET, and GLI) when compared with the DC group (p < 0.001). Whereas statistically significant decreased levels of SGPT and bilirubin are observed in PHC 250 and PHC 500 groups when compared with MET and GLI groups (p < 0.001) (Table 4, Figure 6).

Histopathological examination of the effect of PHC 250 on liver tissue

PHC 250 Liver

**Figure 7:**
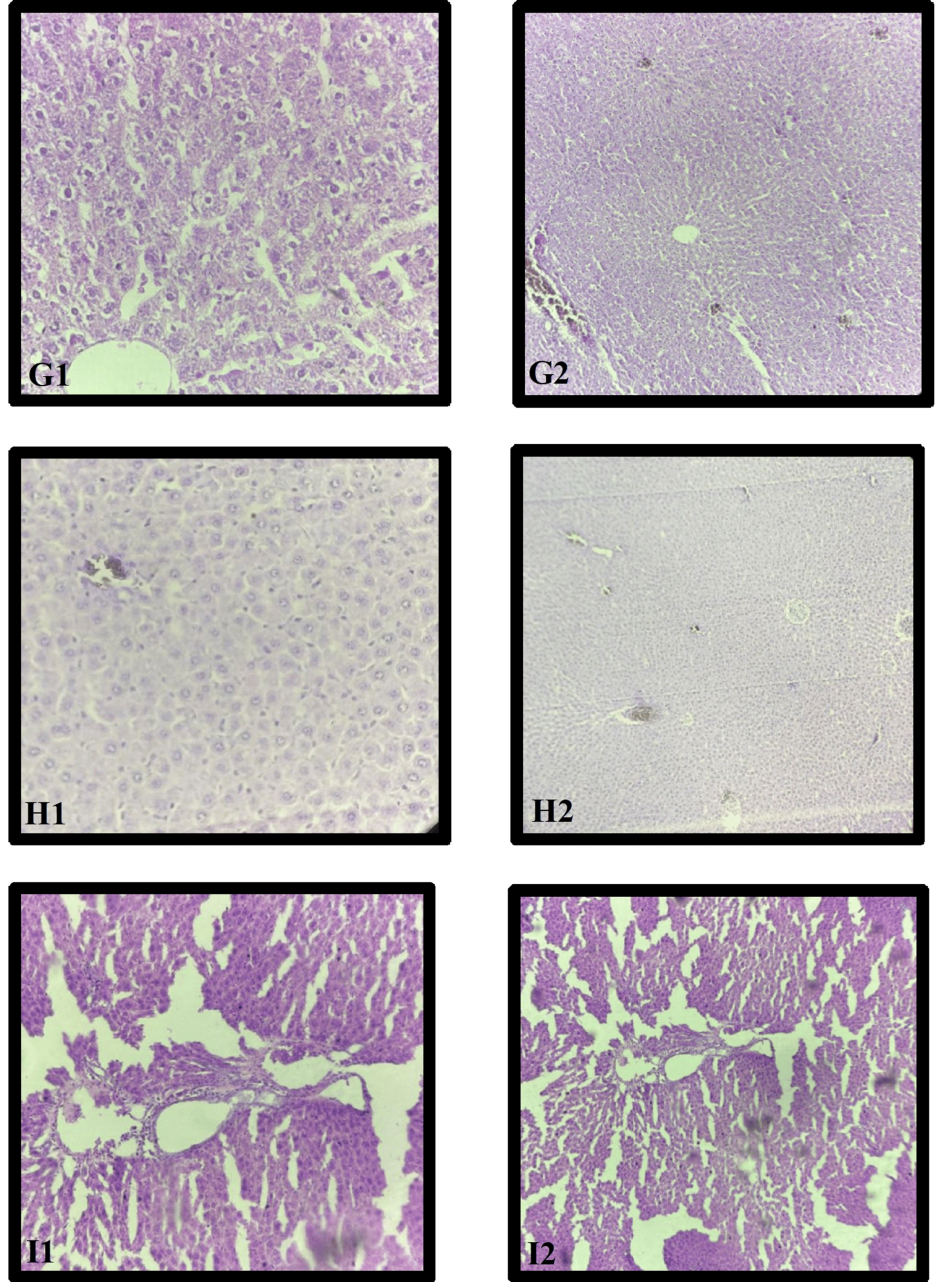
Histopathological examination of the effect of PHC in different doses on liver tissue G: PHC 250; H: PHC 500; I: PHC 1000 1: magnification of 40×; 2: magnification of 10×

Histopathological analysis of the liver cells in the PHC 250 group revealed synovial inflammation and ballooning degeneration of cells, as observed in the photomicrograph under both 10× (G2) and 40× (G1) magnifications (Figure 7).

PHC 500 Liver

Histopathological analysis of the liver cells in the PHC 250 group revealed synovial inflammation and ballooning degeneration of cells, as observed in the photomicrograph under both 10× (G2) and 40× (G1) magnifications (Figure 7).

PHC 1000 Liver

Histopathological analysis of the liver cells in the PHC 1000 group revealed the normal architecture of the central vein, peripheral vein, and hepatocytes, comparable to that of the NC group, as observed in the photomicrograph under both 10× (I2) and 40× (I1) magnifications (Figure 7).

Liver damage scoring results based on histopathological evaluation

Using the semiquantitative liver damage scoring system, histopathological sections from each experimental group were evaluated for hepatocyte degeneration, inflammatory infiltration, vascular congestion, and fibrosis. DC group exhibited severe liver damage (score: 15) with extensive hepatocyte necrosis, heavy inflammation, severe congestion, and bridging fibrosis.

BA and WS treatments showed dose-dependent hepatoprotection, with higher doses (1,000 mg/kg) fully restoring normal liver architecture (score: 0). PHC treatment (500 and 1,000 mg/kg) demonstrated the best hepatoprotective effect, with complete normalization of liver structure at higher doses (score: 0).

Standard drugs (MET and GLI) showed partial protection, with mild hepatocyte swelling and minimal inflammation remaining (score: 4). Overall, PHC (1,000 mg/kg) was the most effective preventive agent for diabetes-induced liver damage, closely followed by BA (1,000 mg/kg) and WS (1,000 mg/kg).

The results confirm that BA, WS, and their combination (PHC) exhibit strong hepatoprotective effects, particularly at higher doses (1,000 mg/kg). The PHC outperformed individual treatments, suggesting a synergistic effect in protecting against diabetes-induced liver injury (Table 5).

**Table 5:** Liver damage scoring NC: normal control; DC: diabetic control; BA: *B. asiatica*; WS: *W. somnifera*; PHC: polyherbal combination; MET: metformin; GLI: glimepiride

Group	Hepatocyte degeneration	Inflammatory infiltration	Vascular congestion	Fibrosis/collagen deposition	Total score (severity index)
NC	0 (normal hepatocytes)	0 (no inflammation)	0 (no congestion)	0 (no fibrosis)	0 (normal liver structure)
DC	4 (extensive necrosis)	4 (heavy inflammatory infiltrates)	4 (severe congestion and hemorrhage)	3 (bridging fibrosis)	15 (severe liver damage)
BA 250 mg/kg	2 (moderate vacuolization)	2 (moderate inflammation)	2 (moderate congestion)	1 (mild fibrosis)	7 (mild-moderate damage)
BA 500 mg/kg	1 (mild hepatocyte swelling)	1 (scattered inflammation)	1 (mild congestion)	1 (mild fibrosis)	4 (minimal liver damage)
BA 1000 mg/kg	0 (normal hepatocytes)	0 (no inflammation)	0 (no congestion)	0 (no fibrosis)	0 (normal liver structure)
WS 250 mg/kg	2 (moderate vacuolization)	2 (moderate inflammation)	2 (moderate congestion)	1 (mild fibrosis)	7 (mild-moderate damage)
WS 500 mg/kg	1 (mild hepatocyte swelling)	1 (scattered inflammation)	1 (mild congestion)	1 (mild fibrosis)	4 (minimal liver damage)
WS 1000 mg/kg	0 (normal hepatocytes)	0 (no inflammation)	0 (no congestion)	0 (no fibrosis)	0 (normal liver structure)
PHC 250 mg/kg	1 (mild vacuolization)	1 (mild inflammation)	1 (mild congestion)	1 (mild fibrosis)	4 (minimal liver damage)
PHC 500 mg/kg	0 (normal hepatocytes)	0 (no inflammation)	0 (no congestion)	0 (no fibrosis)	0 (normal liver structure)
PHC 1000 mg/kg	0 (normal hepatocytes)	0 (no inflammation)	0 (no congestion)	0 (no fibrosis)	0 (normal liver structure)
MET	1 (mild hepatocyte swelling)	1 (scattered inflammation)	1 (mild congestion)	1 (mild fibrosis)	4 (minimal liver damage)
GLI	0 (normal hepatocytes)	0 (no inflammation)	0 (no congestion)	0 (no fibrosis)	0 (normal liver structure)

## Discussion

Liver function is essential in maintaining overall metabolic balance, detoxification, and biochemical synthesis in the body [13]. The rising incidence of liver disorders globally has led to increased interest in alternative therapies that utilize natural products. BA and WS are two herbs with promising hepatoprotective properties [14]. BA is known for its anti-inflammatory and antioxidant effects [15], while WS is widely recognized for its adaptogenic and antioxidant capabilities [16]. This study evaluates the effects of these herbs, individually and in combination, on liver function in rat models, providing insights into their potential use as natural hepatoprotective agents.

This research addresses a critical gap in the existing literature by exploring the combined hepatoprotective effects of BA and WS in a diabetic model. Given the rising interest in natural therapies, the findings of this study could pave the way for the development of effective, plant-based interventions for managing diabetes-associated liver dysfunction. Furthermore, understanding the synergistic interactions between these herbs may offer new insights into multitargeted therapeutic strategies for complex metabolic disorders.

The study demonstrated that both BA and WS, individually and in combination, significantly improved both liver function markers, i.e., SGPT and bilirubin levels, compared to the DC group. Significant reductions in SGPT levels were observed across all treatment groups (BA 250, BA 500, BA 1000, WS 250, WS 500, WS 1000) when compared to the DC group (p < 0.001). The combination therapy (PHC 250, PHC 500, PHC 1000) exhibited even greater reductions, especially at higher doses. Similarly, bilirubin levels were significantly reduced in all treatment groups compared to the DC group (p < 0.001), with the combination therapy showing the most substantial effect.

The combination of BA and WS (PHC) in our study demonstrated a more pronounced effect on reducing these markers, indicating a potential synergistic effect. The PHC showed greater efficacy in reducing liver enzymes compared to individual treatments. This is consistent with research by Leena et al., who observed enhanced therapeutic outcomes when combining different herbal extracts for liver protection. This suggests that such combinations could offer a more effective approach to managing liver health [17].

Histopathological analysis of liver tissue

Liver photomicrographs from the NC group displayed intact hepatic architecture. Central and peripheral veins maintained structural integrity, and hepatocytes showed consistent morphology without signs of congestion, inflammation, or degeneration.

Liver sections from the DC group revealed marked pathological changes characterized by severe vascular congestion in the central vein and extensive ballooning degeneration of hepatocytes. These changes suggest significant hepatic damage and cellular distress. Lower doses of BA and WS induced mild vascular congestion in the central vein. PHC-treated groups (250 and 500 mg/kg) exhibited synovial inflammation and hepatocyte ballooning, indicating moderate hepatocellular injury.

Liver tissues from 1,000 mg/kg BA, WS, and PHC groups preserved normal hepatic architecture, closely aligning with NC profiles. Central and peripheral veins appeared intact, and hepatocytes were structurally sound. MET-treated liver samples showed mild vascular congestion, whereas GLI-treated liver tissues displayed normal hepatic architecture, reflecting preservation of liver integrity similar to the NC group.

The hepatoprotective effects of our study coincide with a few other studies done by researchers like Zuo et al. and Tiwari and Khosa on both herbs individually [18,19]. For instance, BA has been shown to reduce liver enzyme levels and oxidative stress markers in various studies previously reported by Jahan et al. [20]. Similarly, Gupta and Rana have documented that WA has demonstrated hepatoprotective effects through its antioxidant and adaptogenic properties [21]. The findings of this study are consistent with these reports, further validating the hepatoprotective properties of these herbs. However, the novel aspect of this study lies in evaluating their combined effect, which has not been extensively studied. The combination therapy’s enhanced effect on liver function markers suggests a potential synergistic interaction between the two herbs, which could offer superior therapeutic benefits is also studied by Mishra et al. [22].

The hepatoprotective effects observed in this study may be attributed to the active constituents of BA (e.g., berberine) [23] and WA (e.g., withanolides). Berberine is known to exert anti-inflammatory and antioxidant effects by inhibiting proinflammatory cytokines and reducing oxidative stress [24]. Withanolides, on the other hand, enhance antioxidant defenses by upregulating enzymes like superoxide dismutase and catalase [25]. Combining these herbs could potentially target multiple pathways involved in liver protection, leading to the observed synergistic effect.

This study introduces the concept of a synergistic herbal approach to liver function enhancement. The findings suggest that combining BA and WA may offer a more effective strategy for liver protection than using either herb alone. This approach could be explored further in clinical settings, offering a natural, multitargeted therapy for liver disorders.

The significant improvements in liver function markers observed in this study highlight the potential of these herbs as natural hepatoprotective agents. Future studies should focus on clinical trials to validate these findings in human subjects. Additionally, exploring the effects of different dosages and formulations could help optimize the therapeutic potential of these herbs.

Limitations

While this study provides valuable insights, it is limited by its use of animal models, which may not fully replicate human liver pathology. Moreover, the study focuses on specific doses and markers, which may not capture the full spectrum of the herbs' effects. Future research should address these limitations and include a broader range of dosages, longer study durations, and clinical trials.

## Conclusions

This study highlights the hepatoprotective potential of BA and WS in STZ-NIC-induced T2DM Wistar rat models. Both herbs individually showed significant hepatoprotective effects. They reduced SGPT and bilirubin levels, which are markers of liver function, indicating their protective role in mitigating liver dysfunction. The combination of BA and WS in equal proportions (PHC) demonstrated a superior protective effect compared to the individual treatments. Higher doses of PHC (1,000 mg/kg) were especially effective, yielding results comparable to standard drugs like MET and GLI. Histopathological findings also support that PHC at higher doses preserved the normal architecture of liver tissues, reducing signs of vascular congestion, inflammation, and hepatocyte ballooning observed in DCs.

The study highlights the potential synergy between BA and WS, as the combined treatment exhibited enhanced efficacy compared to individual treatments. The findings underscore the potential of BA and WS as cost-effective, natural alternatives for managing diabetes-induced liver damage. This supports the broader use of herbal combinations in therapeutic settings. PHC of BA and WS could be a viable strategy for mitigating diabetes-associated hepatic dysfunction.

Clinical studies are recommended to validate these findings in humans. Additionally, exploring various dosages and formulations could help optimize therapeutic potential.
